# Diverse Roles of Heparan Sulfate and Heparin in Wound Repair

**DOI:** 10.1155/2015/549417

**Published:** 2015-07-07

**Authors:** Pawel Olczyk, Łukasz Mencner, Katarzyna Komosinska-Vassev

**Affiliations:** ^1^Department of Community Pharmacy, Faculty of Pharmacy with the Division of Laboratory Medicine, Medical University of Silesia, Kasztanowa 3, 41-200 Sosnowiec, Poland; ^2^Department of Clinical Chemistry and Laboratory Diagnostics, Faculty of Pharmacy with the Division of Laboratory Medicine, Medical University of Silesia, Jednosci 8, 41-200 Sosnowiec, Poland

## Abstract

Heparan sulfate (HS) and heparin (Hp) are linear polysaccharide chains composed of repeating (1*→*4) linked pyrosulfuric acid and 2-amino-2-deoxy glucopyranose (glucosamine) residue. Mentioned glycosaminoglycans chains are covalently *O*-linked to serine residues within the core proteins creating heparan sulfate/heparin proteoglycans (HSPG). The latter ones participate in many physiological and pathological phenomena impacting both the plethora of ligands such as cytokines, growth factors, and adhesion molecules and the variety of the ECM constituents. Moreover, HS/Hp determine the effective wound healing process. Initial growth of HS and Hp amount is pivotal during the early phase of tissue repair; however heparan sulfate and heparin also participate in further stages of tissue regeneration.

## 1. Introduction

Wound healing, physiological body response to injury, is a complex series of events leading to the repair of damaged tissues and reestablishment of cellular homeostasis. These dynamic biochemical pathways involve four overlapping but well-defined phases: haemostasis, inflammation, proliferation, and remodeling [1, 2]. The restoration of tissue integrity requires precise cooperation of many cells such as neutrophils, macrophages, fibroblasts, and epithelial and endothelial cells, interacting with one another and with the components of the extracellular matrix (ECM) through their integrin receptors and adhesion molecules. In addition to various cellular interactions, wound repair process is tightly regulated by different cytokines, growth factors, and proteolytic enzymes which create balanced wound molecular environment necessary for continuing effective healing [3–6]. Matrix molecules including glycosaminoglycans (GAG) play an essential role in wound repair activity through all phases of the healing process. The GAG family consists of sulfated glycosaminoglycans, that is, chondroitin/dermatan sulfate (CS/DS), heparan sulfate/heparin (HS/Hp), and keratan sulfate (KS), as well as unsulfated hyaluronic acid (HA). The first three types of molecules are covalently connected to core protein forming proteoglycans (PGs). Hyaluronic acid does not form covalent links with proteins but instead interacts noncovalently with proteoglycans via hyaluronan-binding motifs [7, 8]. GAGs influence wound healing process by providing both a scaffold support and a signaling role. ECM components create a temporary matrix in the repairing process [6, 9]. Signal transduction role is being fulfilled by stimulation of cellular adhesion, migration, differentiation, and proliferation as well as regulation of ECM organization and metabolism. Mentioned functions are connected with GAGs and PGs ability to bind with high affinity to a range of cytokines, growth factors, and members of chemokine superfamily. These interactions additionally can serve as a reservoir of regulatory factors that can be liberated by selective degradation of GAG chains [10, 11]. PGs may also have other roles in wound healing including a direct influence on inflammation [2]. Moreover, extensive changes in ECM components in the course of repair process may be reflected in reepithelialization and regeneration of the basement membrane but also may influence intercellular communication [12]. Heparan sulfates have diverse functions with respect to skin wound healing. A large chemical diversity of HS chains and capacity of these glycosaminoglycans to interact with proteins and diverse binding ligands through the varied arrangements of sulfate groups and glucuronic acid/iduronic acid residues determine their contribution to effective tissue repair. Understanding the complex mechanisms by which these ECM components influence wound repair activity promises the implementation of new therapeutic strategies.

## 2. Structure and Properties of Heparan Sulfate and Heparin

**Figure 1:**
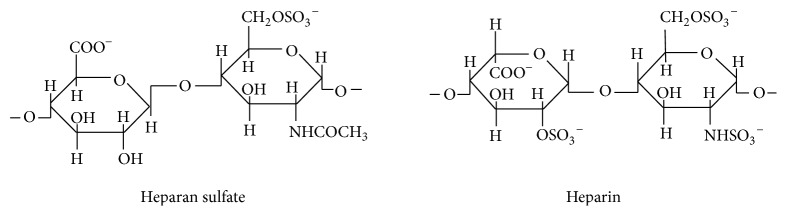
Structure of heparan sulfate/heparin disaccharides [13].

Heparan sulfate (HS) and heparin (Hp) are the glycosaminoglycans (GAGs) of the most complex structure among all GAGs. They are made of recurring, disaccharide units consisting of glucuronic acid and N-acetylglucosamine residues of a schematic structure [→4GlcA*β*1→GlcNAc*α*1→], in which the glycosidic bond between the hexuronic acid and N-acetylglucosamine assumes the configuration of *β*1→4 instead of *β*1→3, while the bond between N-acetylglucosamine and hexuronic acid assumes the configuration c*α*1→4 instead of *β*1→4, as it takes place in chondroitin-dermatan glycosaminoglycans [11, 13–16]. HS/Hp structures are presented in Figure 1.

Despite the fact that heparin is often considered an analogue of heparin sulfate, which is caused by the fact that both GAGs are made of the same, monomeric subunits, postsynthetic modifications, the range of which is significantly different in both glycosaminoglycans, definitely differentiate these biopolymers [17]. Namely, monomeric heparin subunits are sulfated to a greater degree than the subunits of HS [13]. On average, one disaccharide unit of heparin contains 3 sulfate groups, while one disaccharide unit of heparan sulfate contains only one sulphate group [18]. The negative charge density, which is displayed by heparin, is the highest among biologic macromolecules and is responsible for the fact that this GAG is the most acidic macromolecule of human body [7, 19, 20].

Iduronic acid dominates in heparin structure constituting 90% of all acid residues, while in the heparan sulfate, glucuronic acid, being the C5 epimer of the iduronic acid [18, 21], occurs in greater amounts.

The molecular mass of the heparin molecule on average equals 15 kDa, while in the case of heparin sulfate about 30 kDa. Moreover, the chains of the latter one are longer than in the case of heparin [14].

The molecules of heparin sulfate are characterized by a greater heterogeneity of the structure compared with the structure of heparin. HS contains bigger amounts of the acetylated glucosamine residues than N-sulfated GlcN, greater content of GlcA than IdoA, but fewer O-bound sulfate groups. Moreover, HS displays the domain structure comprising highly sulfated, heparin-like sequences, poorly sulfated sequences, and unmodified regions: [GlcA-GlcNac] [14, 18, 19, 22, 23].

## 3. Heparan Sulfate and Heparin Biosynthesis, Postsynthetic Modification, and Degradation

The two kinds of GAGs also differ with regard to the tissue location, core proteins, to which, in the process of biosynthesis, glycans are linked, and the number of glycan chains connected with the protein [13]. Heparin is synthesized in the mast cells and basophils, in the form of side, glycosaminoglycan chains of the proteoglycan, serglycin [18, 24]. This proteoglycan, which contains numerous glycan chains of uneven length, is secreted from the mentioned cells in the process of their degranulation, after which the enzymatic degradation takes place with a subsequent release of heparin [18]. Heparan sulfate is also synthesized as a proteoglycan component, which is a constituent of many PGs occurring on the cell membrane or located in the extracellular matrix [18, 25]. Syndecans and glypicans are the two main families of HSPG which are located on the cell surface [26–28]. Moreover, perlecan, agrin, and collagen type XVIII also belong to this HSPG family and, furthermore, the isoform CD44, betaglycan, and testican [17, 29] which constitute not more than over 5% of all heparan sulfate PGs. On average, HSPG consists of only a few HS chains [17, 18].

The initial biosynthesis stages of heparan sulfate proteoglycans are not different from the initial biosynthesis stages of CS/DSPG. The linking tetrasaccharide region, connected with the seryl residue of the core protein, initiates the elongation of the HS/Hp chain [14]. In this process, the monosaccharide subunits, the N-acetylglucosamine and glucuronic acid, are alternately linked to the nonreducible end of the growing glycan chain by the glycosyltransferases, the so-called exostosin I (EXT1), and exostosin II (EXT2) [30]. During the polymerization, the glycan chain is subject to many modifications [14]. These modifications concern N-deacetylation and N-sulfation of glucosamine, epimerization of GlcA to IdoA, 2-O-sulfating of hexuronic acid (usually IdoA), 6-O-sulfating, and 3-O-sulfating of glucosamine. They start from the removal of N-acetyl groups, after which they are replaced by the sulfate groups. The latter process is catalyzed by the enzymatic complex of N-deacetylase/N-sulfotransferase (NDST) [29]. N-deacetylation and N-sulfation are the initial condition of all next, enzymatic modifications [30]. Next to them is the transformation of glucuronic acid into iduronic acid, catalysed by C-5 epimerase, which is specific for both HS and Hp [14].

Similarly, the activity of 2-O-sulfotransferase (HS2ST) displays the specificity towards HS and Hp. This enzyme catalyses the sulfating reaction of hexuronic acid. It is thought that both last enzymes create a complex or enter the interaction in order to reverse epimerization, already sulfated IdoA back into GlcA [30]. Another enzyme in the modification process of the increasing HS/Hp chain 6-O-sulfotransferase heparin sulfate (HS6ST) transfers 6-O-sulfate group onto N-sulfated glucosamine, while heparan sulfate 3-O-sulfotransferase transfers 3-O-sulfate group also onto the residue of the mentioned hexosamine.

The modifications of heparan sulfate proteoglycans also comprise, besides the ones connected with the biosynthesis of the glycan chain, the ones concerning the transformation of the HSPG core protein [30].

HSPG comprise the syndecan family, transmembrane PGs created from 4 members- syndecan-1, -2, -3, -4, sometimes “enriched” in CS chains; glypican family PGs bound with the cell membrane by glycosylphosphatidylinositol having six members (glypican-1,-2,-3,-4,-5,-6); betaglycan PG of the cell surface, also named the type III receptor for TGF-*β*; testican (chondroitin-heparan sulfate PG of extracellular matrix); perlecan; and agrin (PGs of basement membranes) [23, 25, 29, 31–34]. Perlecan may also occur outside the basement membranes, that is, in the extracellular matrix of tissues deprived of these structures, as in the case of the matrix of cartilage, sinusoid vessels of liver, spleen, or in the lymph nodes [35].

It is worth mentioning that proteins may also undergo a modification by binding HS chains which, however, usually does not occur in this kind of proteins. Such examples are isoform CD44 or collagen type XVIII [30].

Another postsynthetic modification of HSPG is the release of GAG chains from their proteoglycans (the so-called “shedding”), which leads to the transformation of insoluble glycans, connected with this protein, into free, soluble form of these compounds. These soluble GAGs may undergo further, enzymatic transformations, which are connected with the change of the length of glycan chain or with “revealing” specific domains (masked up to this moment). A special role in the mentioned enzymatic transformations is played by heparanase (endoglucuronidase), degrading the HS chains [30, 36, 37].

The degradation of heparan sulfate proteoglycans initially takes place in the extracellular space, and then is continued in lysosomes. Heparanse (endo-beta-glucuronidase) is endoglycosidase, specific for heparan sulfates, degrades the abovementioned glycans to smaller fragments (in the place of glucuronide bonds) [30, 38].

## 4. Heparan Sulfate and Heparin Physiological Functions

Biological functions of heparan sulfate glycosoaminoglycans have been well recognized. Heparan sulfate plays a significant role in regulating the interactions between cells and between cells and extracellular matrix. HS stimulates the adhesion of cells to ECM by binding themselves to matrix macromolecules such as fibronectin or laminin [18, 39, 40]. HS and Hp function as endogenous receptors for numerous extracellular ligands, growth factors, and chemokines which all regulate the process of cell proliferation, differentiation, and angiogenesis, processes of leukocytic migration and degranulation, and processes of carcinogenesis [14, 26, 27, 35, 41–45]. The role of HSPG in metastases depends on the type of the tissue, pathophysiologic condition of the cancer and on the HSPG tissue location. Generally, although not always, the HSPG of the cell surface prevent metastases, while the core proteins and HS chains themselves modulate the mentioned process [37]. Heparin, synthesized in basophils and mast cells of lungs, intestines, and liver, plays a role in the body immune defenses. It demonstrates antioxidant, anti-inflammatory and vasodilating properties [19]. Together with heparan sulfate, it demonstrates anticoagulant activity; however, in vivo the function of Hp in the regulation of the coagulation process seems to be very unlikely [24, 46, 47]. The HSPG of cell surface regulate the metabolism of lipoproteins [33]. The participation of HSPG in the processes of neurogenesis and repair of tissue damage is known [11, 46–50].

## 5. The Role of Heparan Sulfate and Heparin in the Process Wound Healing

Initial growth of HS/Hp amount is pivotal during early stages of tissue repair. It is known that HS/Hp play a key role in chemical signaling between cells through binding and regulating the activities of heparin-binding growth factors, proteolytic enzymes, and protease inhibitors [51, 52].

Heparin impacts hemostatic phase of wound healing by binding of various molecules. Mentioned glycan interacts with antithrombin participating in serpin's coagulation cascade, proteinase nexin-1 functioning as an inhibitor of trypsin-like serine proteases, protein C inhibitor, which plays a procoagulant role, and factors (IIA, IXa, and Xa) taking part in coagulation cascade of serine proteases [53]. Moreover, heparin acts as a potent anti-inflammatory agent that inhibits enzymes and cytotoxic mediators, released from proinflammatory cells, responsible for augmentation of inflammation, such as elastase, cathepsin G, eosinophil peroxidase, eosinophil cationic protein, major basic protein, interleukin-8, and stromal-derived factor-1 [53, 54]. On the other hand, heparan sulphate enhances the recruitment of inflammatory cells, since endothelial surface HS decreases neutrophil rolling rapidity via L-selectin mediated cell adhesion. Moreover, HS-mediated Mac-1-CD44v3 interaction stimulates binding of leukocytes to the endothelial surface to drive the cells extravasation [29]. Last but not least, HS can be recognized as a sensor of tissue injury, thanks to the interaction with TLR-4 on leukocytes. This action regulates the release of proinflammatory cytokines by macrophages and significantly enhances the maturation of dendritic cells. Mentioned phenomenon is confirmed by the upregulation of MHC-II, CD40, ICAM-1, CD80, CD86, and reduced antigen uptake [29]. HS/Hp are recognized as pivotal players in angiogenesis, cell growth, migration, and differentiation [51, 55, 56].

HS/Hp, abundant in acute wound fluid 24–72 h after injury, bind heparin binding growth factor (Hb-EGF), which can act as a mitogenic agent for fibroblasts, smooth muscle cells, and epithelial cells [57]. Moreover, after skin damage, heparan sulfate proteoglycan, syndecan-4, is upregulated within the granulation tissue on fibroblasts and endothelial cells, which may suggest that syndecan-4 regulates wound healing and related angiogenesis [58].

HS/Hp interact with hepatocyte growth factor, which regulates cell growth, motility, and morphogenesis of epithelial or endothelial cells and stimulates epithelial repair and neovascularization [46, 53, 59]. HS/Hp also influence fibroblast growth factor responsible for cell proliferation, differentiation, signal transduction, and angiogenesis [46, 53].

The presence of heparin at high concentrations reduces the activity of FGF-7, [60] which is responsible for enhancement of keratinocytes migration and proliferation and plays a key role in reepithelialization process [61]. The mentioned heparin “conditions” do not inhibit the action of another important factor [60], that is, FGF-1, which regulates the proliferation of fibroblasts, endothelial, and epithelial cells and influences angiogenesis via effect on the activity of endothelial cells [62]. Special attention should be paid to the fact that heparin can enhance the stability of FGF1 and may determine the formation of FGF1-FGFR (fibroblast growth factor receptor) active complex [63]. The heparin's small fraction presents high affinity to FGF-7, particularly supporting the FGF7/FGFRIIIb signaling [60]. Furthermore, HS, which builds the heparan sulfate proteoglycan, that is, syndecan-1, binds FGF-7 and its receptor, promotes the FGF-7 signaling and influences organization of granulation tissue. However, the overexpression of syndecan-1 may modify the HS effect, from stimulatory into inhibitory one, on FGF-7 signaling [60].

Last but not least, it should be noted that heparan sulfate may be responsible for accurate regulation of wound angiogenesis through binding and modulation of various paracrine agents, such as VEGF, FGF, TGF-*β*, PDGF-*β*, SDF-1, and MCP-1, functioning in orchestrated and interactive mode [58]. VEGF-A is a master regulator of angiogenesis influencing various aspects of the mentioned phenomenon, including endothelial cells differentiation, assembly, proliferation, or migration [58]. FGF-1,-2 promotes endothelial cell proliferation and the physical organization of endothelial cells into tube-like arrangements [62]. TGF-*β* may participate in vessel stabilization and quiescence, since the components of the TGF-*β* signaling pathway, including TGF-*β* receptors, interact and cocluster directly with VE-cadherin at EC-EC junctions [58]. PDGF-*β* signaling is crucial for mural cells recruitment, vascular maturation, and stability [58]. The chemokine stromal-cell-derived factor-1 (SDF-1) inhibits human microvascular endothelial cells apoptosis and enhances cell proliferation and capillary tube formation [64]. Monocyte chemoattractant protein-1 (MCP-1) regulates the angiogenic effect of TGF-*β* by recruiting vascular smooth muscle cells and mesenchymal cells toward endothelial cells [65].

Moreover, the morphology of syndecan-1-null wounds was reported to be more changeable, but the reepithelialized epidermis was organized in a lesser extent and was thinner than in the case of the control ones indicating a possible role for mentioned HSPG in the signaling mediation or in remodelling the recently laid dermis [60].

HS/Hp, which interact with TGF-*β*1 and potentiate its activity, are indispensable for adhesive and contractile signaling, that results in myofibroblast formation and wound closure [51, 66, 67].

In our previous experimental studies, we proved that glycosaminoglycans, including heparan sulfate/heparin, chondroitin/dermatan sulfates, and hyaluronic acid, turned out to be better effectors of natural therapeutic agent such as propolis than silver sulfadiazine (agent of choice in local burn management) in animal burn wound healing model [51, 68]. Moreover, our studies have shown the beneficial effect of propolis on the other extracellular matrix constituents, that is, collagens, fibronectin, laminin, and vitronectin, remodeling in burnt skin. Propolis, as a factor modulating the expression of the collagens, noncollagenous proteins, and glycosaminoglycans, speeds up the healing process and contributes to scar-less healing of the burnt skin [51, 69, 70]. The strong positive effect of propolis on decreasing the amount of free radicals, the factors playing an important role in the postsynthetic modification of the ECM components, was proved in our earlier study concerning burn wound healing [71].

In conclusion, understanding biochemical changes of the ECM constituents proceeding with healing process may be of great importance in the implementation of the new alternative therapeutic strategies, in the course of thermally damaged tissues repair.
